# The Effect of a WhatsApp Chatbot‐Based Training Program on Infection Control Knowledge and Practices Among Hemodialysis Nurses: A Cluster‐Randomized Controlled Trial

**DOI:** 10.1155/jonm/2281714

**Published:** 2026-05-17

**Authors:** Hossam Alhawatmeh, Seba M. Issa, Ibrahim Alasqah

**Affiliations:** ^1^ Department of Adult Health Nursing, College of Nursing, Jordan University of Science and Technology, Irbid, 44220, Jordan, just.edu.jo; ^2^ Department of Community, Psychiatric and Mental Health Nursing, College of Nursing, Qassim University, Buraydah, 52571, Saudi Arabia, qu.edu.sa

**Keywords:** artificial intelligence, distance, education, infection control, knowledge, nurses, practice patterns, renal dialysis

## Abstract

**Background:**

Patients undergoing hemodialysis are highly susceptible to infection; however, evidence suggests variability in nurses’ infection control competencies. Although digital learning tools are increasingly used in nursing, there have been few randomized controlled trials assessing WhatsApp chatbot‐based infection control training for hemodialysis nurses. Thus, the purpose of this study was to assess the effect of a WhatsApp chatbot‐based training program on nurses’ knowledge and practices related to infection control in hemodialysis units.

**Methods:**

A cluster‐randomized controlled trial was conducted from July to September 2024, involving 50 hemodialysis nurses recruited via convenience sampling from five hospitals in Jordan. Ten clusters, defined by hospital site and work shift, were randomly assigned using a computer‐generated sequence prepared by an independent researcher to either an intervention group, which received the WhatsApp chatbot‐based training program, or a control group. Guided by constructivist learning theory, the intervention facilitated active knowledge construction through an interactive, automated chatbot dialogue delivered over 4 weeks. Outcome measures were collected using a self‐reporting questionnaire at baseline and 4 weeks after completion of the 5‐day intervention.

**Results:**

Mixed‐model repeated**-**measures ANOVA revealed that the intervention group showed statistically significant improvements in both knowledge (*F* (1, 48) = 19.34, *p* < 0.0001, *η* ^2^ = 0.28) and practice (*F* (1, 48) = 14.52, *p* < 0.0001, *η* ^2^ = 0.23), both indicating large effect sizes, compared with the control group.

**Conclusion:**

The study results indicate that the WhatsApp chatbot‐based training program is an effective e‐learning approach that helped hemodialysis nurses develop their infection control knowledge and practice. The results suggest that WhatsApp chatbot‐based education provides nurse managers with a flexible and scalable method to enhance infection control competencies and improve patient safety in hemodialysis units.

## 1. Introduction

End‐stage renal disease (ESRD) is the terminal stage of chronic kidney disease (CKD), which affects 9.1%–13.4% of the global population—approximately 700 million to one billion people [1]. In Jordan, the number of patients with ESRD reached 7747 during 2020, which led to 691 deaths [2]. Hemodialysis is the primary treatment, and according to the Jordan Renal Registry, 97.6% of patients in Jordan received hemodialysis in 2020 [2]. While life‐sustaining, hemodialysis inherently elevates the risk of healthcare‐associated infections (HAIs). This vulnerability is primarily driven by frequent vascular access, patient‐related immunodeficiency, and suboptimal staff adherence to standardized infection control protocols. HAIs are a leading cause of death among patients receiving hemodialysis worldwide, including in Jordan [2–5].

Strict adherence to infection control protocols is paramount for healthcare professionals in hemodialysis units to mitigate the risk of HAIs [6]. However, a critical implementation gap persists; research indicates that hemodialysis nurses, particularly in Jordan, exhibit insufficient knowledge and suboptimal infection control practices [7–9]. The implementation of continuous professional development training serves as a vital approach to enhance nurses’ infection control competencies, which results in decreased patient mortality and illness severity [5, 10, 11]. The previous ongoing professional development program has largely relied on traditional face‐to‐face training methods as its standard teaching approach. Studies using quasiexperimental methods demonstrated that nurses who received face‐to‐face training developed better infection control knowledge and skills in Egyptian pediatric hemodialysis units. However, the studies had several limitations, including the use of a small sample, a single setting, and a single group, which reduced their validity and the ability to generalize the results to different study groups [5, 12, 13]. It has also been reported that these training models encounter multiple operational difficulties because they require expensive operations and struggle to retrieve nurses who need development training from their assigned duties [14, 15].

To overcome the operational and scheduling barriers associated with traditional face‐to‐face training, e‐learning has emerged as an essential approach, particularly through mobile learning (m‐learning). While m‐learning improves accessibility by enabling learners to engage with content anytime and anywhere, recent evidence suggests that mobile‐based interventions can effectively improve nurses’ knowledge, attitudes, and practices related to infection control in clinical settings [16]. However, its effectiveness can still be limited by technological barriers, especially when it requires the use of unfamiliar applications or lacks sufficient interactivity [17, 18].

Advances in artificial intelligence have introduced more interactive approaches to nursing education, shifting learning from passive information delivery to more engaging and guided experiences. AI‐driven tools have been shown to enhance knowledge acquisition, clinical reasoning, and learner engagement, particularly in complex and high‐acuity clinical settings [18–20]. Among these, chatbot technology provides a practical solution by enabling structured, interactive learning with immediate feedback while remaining accessible to nurses who cannot leave their clinical duties [19, 21–25]. However, many chatbot‐based interventions rely on standalone platforms that may disrupt clinical workflows or require additional technical adaptation [17, 19].

WhatsApp offers a familiar and widely used platform that minimizes these barriers [26, 27], although its use as a basic messaging tool lacks the structure required for complex clinical training [17–20]. Integrating chatbot functionality within WhatsApp combines accessibility with structured learning, offering a practical and scalable approach for delivering standardized training in complex, high‐acuity environments such as hemodialysis units [26].

Despite the effectiveness of digital education and the widespread familiarity of Jordanian nurses with WhatsApp [12, 16, 23, 24], an important research gap remains. Previous studies have primarily used WhatsApp as a communication tool to disseminate educational materials—such as text‐based content, clinical guidelines, PDF documents, and discussion messages—to healthcare professionals, including nurses, medical trainees, and students, mainly within general clinical and educational settings. These approaches typically rely on passive, group‐based interactions and are not specifically designed for structured clinical training [17, 18]. In contrast, chatbot‐based interventions in healthcare education have been implemented among nurses, medical trainees, and students, often within academic or general clinical contexts, but are typically delivered through standalone platforms, which may limit accessibility and integration into routine clinical workflows [19, 20, 22–25]. While these approaches have demonstrated potential benefits, they remain limited by their reliance on either passive messaging or standalone chatbot systems and have not integrated structured, interactive chatbot learning within widely used messaging platforms or been evaluated in specialized, high‐acuity settings [28, 29].

In the hemodialysis context, to the best of our knowledge, only one study has implemented a WhatsApp chatbot‐based intervention, focusing on a single aspect of infection control practice—antimicrobial resistance awareness among nurses [26]. To date, no randomized controlled trials have evaluated a comprehensive, interactive infection control training program delivered through a WhatsApp‐integrated chatbot in this specialized setting. Building on this gap, the present study evaluates a constructivist‐based WhatsApp chatbot designed to deliver structured, scenario‐based learning with immediate personalized feedback. Accordingly, this RCT aimed to examine its effects on infection control knowledge and practices among Jordanian hemodialysis nurses. Theoretically, this study contributes to the e‐learning literature by integrating chatbot‐driven instruction within a familiar mobile platform [30, 31], while practically offering a scalable and accessible model for continuous professional development without disrupting clinical workflow [24, 25].

## 2. Method

### 2.1. Study Design

The study employed a pretest–posttest cluster‐randomized controlled design, which took place from July to September 2024. While the participating hospitals were selected via convenience sampling based on geographic accessibility and administrative approval, the allocation of participants followed a strict randomization protocol to maintain methodological rigor. Fifty nurses working in hemodialysis units who met the eligibility criteria and agreed to participate were recruited and assigned at the cluster level (defined by hospital site and work shift) to one of two study groups. The experimental group received education on infection control in hemodialysis units via a chatbot delivered via the WhatsApp application. The control group continued with their usual practices without any additional training.

### 2.2. Study Setting

The study was conducted in a hemodialysis unit of four public hospitals (Princess Basma Teaching Hospital, Muath ben Jabal Hospital, Al‐Iman Hospital, and Al‐Bashir Hospital) and one private hospital (King Abdullah University Hospital). The selection of 4 governmental and 1 private hospital was based on both proportional representation and institutional feasibility. Statistically, the public healthcare sector in Jordan manages the largest share of dialysis infrastructure (47% of machines), making it the primary environment for the nursing workforce [2]. Furthermore, the selection was influenced by the availability of centralized Institutional Review Board (IRB) oversight. While the private sector provides significant care, administrative and ethical approval processes vary significantly across individual private entities; therefore, we prioritized sites with standardized ethical protocols to ensure a timely and uniform start to the intervention.

### 2.3. Sampling and Procedure

The target population of this study was all nurses working at the hemodialysis unit in Jordan. The accessible population was nurses working at the hemodialysis unit in the five study settings. Considering the logistical complexities and rotating work schedules of hemodialysis units involved, convenience sampling methodology was utilized so that an intervention could be effectively implemented into the clinical environment without interfering with the activities of providing the necessary patient care. However, the study potentially achieved better external validity through diverse clinical environment participation despite using convenience sampling. The study took place at five Jordanian hospitals, which operated between the public and private sectors across different city locations. The researchers included different healthcare facilities to create a diverse participant group that would represent the hemodialysis nursing staff across the region.

The inclusion criteria were nurses working in an HD unit, having a smartphone, having at least 6 months of experience in hemodialysis facilities, being able to read and write in English, and being committed to completing all study protocols. Nurses who had received an infection control educational program within the past 6 months were excluded from the study.

**FIGURE 1:**
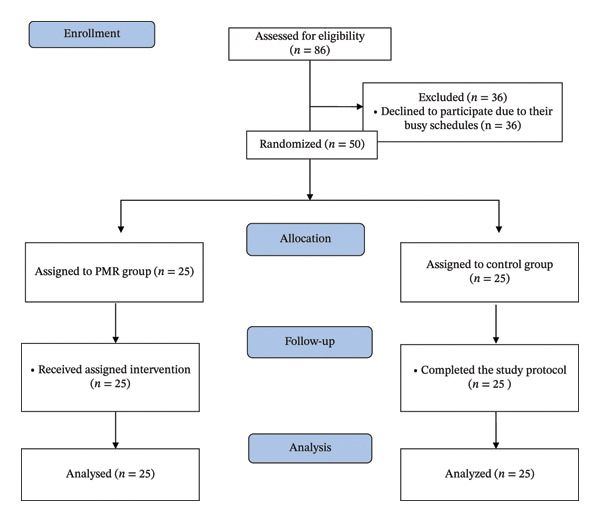
The study flow diagram.

The required sample size was calculated using G∗Power (Version 3.1). Based on a 2 × 2 mixed‐design repeated‐measures (RM) analysis of variance (ANOVA), an effect size of 0.22 derived from pilot data, a significance level of 0.05, and a statistical power of 0.80, the estimated minimum sample size was 44 participants. To account for a potential 10% attrition rate, estimated from a similar study [26], we added 5 participants. An additional 2 participants were included as a conservative buffer, resulting in a final sample size of 50 participants. As shown in Figure 1, a total of 86 nurses were screened for eligibility; however, 36 individuals declined to participate, primarily citing busy clinical schedules and heavy workloads. This introduces the potential for volunteer bias, as the nurses who consented to participate may possess higher levels of baseline motivation or better time‐management capabilities compared to those who declined. Consequently, the study findings might more closely reflect the outcomes of a highly motivated subgroup rather than the entire nursing staff in high‐acuity hemodialysis units.

All 50 enrolled participants (*n* = 25 per group) completed the full study protocol. There was no attrition during the intervention period, and all 50 participants were included in the final analysis (see Figure 1). The 100% completion rate was facilitated by the high accessibility of WhatsApp and the use of real‐time technical support to resolve connectivity issues.

Given the cluster‐randomized design, the potential impact of clustering on statistical efficiency was considered. As no published ICC estimates from studies with comparable populations, clinical settings, and outcomes were identified, a conservative value (ICC = 0.02) was assumed. This is supported by prior literature indicating that ICCs in such contexts are generally small (0.005–0.07) [32]. Using the average cluster size (*m* = 5), the design effect (DE = 1 + (m − 1) × ICC) was 1.08, indicating modest variance inflation.

To further evaluate the robustness of this assumption, sensitivity analyses using ICC values from 0.01 to 0.05 (including 0.02) yielded design effects between 1.04 and 1.20; this range reflects plausible values for behavioral outcomes within the broader reported range [32]. Given the relatively large observed effect sizes (partial *η* ^2^ > 0.20), statistical power was likely maintained (> 0.80) despite the cluster‐based design.

### 2.4. Intervention

The experimental group received a 5‐day self‐paced training program through a customized WhatsApp‐based chatbot, which the researchers built together with a software engineer. The intervention utilized a deterministic conversational agent developed via the ManyChat framework and integrated with the WhatsApp Business API. This system employed rule‐based decision‐tree logic to ensure the standardized delivery of clinical protocols. Through an algorithmic, branching architecture triggered by user keywords and predefined prompts, the agent provided high‐fidelity digital tutoring for multistep sterile techniques. This design was intentionally selected to prioritize clinical safety and procedural accuracy, thereby eliminating the risk of nonstandardized information inherent in nondeterministic systems. Grounded in constructivist learning theory, the program emphasized trainees’ active participation and analytical thinking instead of traditional receipt of information. The research team chose WhatsApp as their delivery platform because it offered free access and was widely used by all Jordanian nurses, resulting in higher adherence [33, 34]. The researchers provided all participants with basic standardized training about chatbot usage before they started the research.

The curriculum content was developed by the research team based on evidence‐based standards from the Centers for Disease Control and Prevention (CDC) [35] and the World Health Organization (WHO) [36]. The program, validated by three experts from diverse fields including medicine, nursing, and infection control, covered five main topics, which included the following:

1. Infections that patients can acquire from hemodialysis.
2. Standard precautions for nurses in all healthcare settings.
3. Specific infection control recommendations for hemodialysis nurses.
4. Basic steps in fistula/graft care.
5. Infection control policies and practices for hemodialysis facilities.

The educational program was structured as a series of five consecutive modular units that were presented on each consecutive morning according to a preset order. In recognition of the tight schedule that the nurses frequently have to face, the researchers carefully designed the everyday interaction process to involve only twenty or thirty minutes of active engagement. The chatbot followed this sequence of daily messages: (1) It began by sending an opening message that showed the assigned topic; (2) it provided educational content through a planned sequence that included text materials, images, video links, and scenario‐based activities; and (3) it concluded with a knowledge assessment that contained 3–5 questions. Participants actively controlled the learning process through self‐paced material advancement, achieved by sending prompts such as “Next” or answer selections to move the conversation thread forward.

The chatbot provided its main educational function through a system that delivered immediate, customized feedback to users. The system provided users with explanations about their mistakes while showing them exactly which learning materials they needed to study again to achieve better results. To ensure the integrity of the study, the experimental group had exclusive access to the intervention through individual WhatsApp accounts. The research team, together with the software engineer, delivered instant technical assistance to handle software problems that emerged during the study duration.

### 2.5. Data Collection Instruments

The researchers created a self‐report questionnaire to assess nurses’ knowledge and practice of infection control measures in hemodialysis units based on CDC [35] and WHO [36] guidelines and current research findings. The identical, validated questionnaire (comprising knowledge and practice sections) was administered at both the baseline (pretest) and postintervention (posttest) measures to ensure reliable, standardized comparisons of change over time. The self‐reported questionnaire was composed of three parts as follows: (1) sociodemographic characteristics, (2) Hemodialysis Nurses’ Infection Control Knowledge Test, and (3) Hemodialysis Nurses’ Infection Control Practice Scale.

### 2.6. Sociodemographic Characteristics

This section included questions about age, gender, educational level, years of experience in the HD unit, and whether the participant had received any previous infection control education program, including the timing of such training.

### 2.7. Hemodialysis Nurses’ Infection Control Knowledge Test

The test involved 33 questions about infection control measures used in hemodialysis, including 13 true/false questions and 20 multiple‐choice questions. The scoring system granted 1 point to the correct answer and 0 to the incorrect answer, resulting in a maximum total score of 33. Higher scores indicated higher levels of knowledge about infection control in hemodialysis.

### 2.8. Hemodialysis Nurses’ Infection Control Practice Scale

The self‐report scale was derived from the CDC public checklist [34, 35], involving 48 items. The scale items were divided into seven parts as follows: arteriovenous fistula/graft cannulation (9 items), hemodialysis catheter connection (8 items), hemodialysis injectable medication preparation (7 items), hemodialysis injectable medication administration (5 items), hemodialysis catheters exit site care (7 items), hemodialysis catheter disconnection (6 items), and arteriovenous fistula/graft decannulation (6 items). The items were rated on a Likert scale, ranging from 1 (*never*) to 5 (*always*). The higher scores reflect better adherence to infection control practices in hemodialysis units.

### 2.9. Validity

The knowledge and practice tools were validated by a panel of three experts in the fields of medicine, infection control, and nursing using a content validity index. Content validity was quantified using the scale‐level content validity index based on the averaging method (S‐CVI/Ave) [37]. The instrument demonstrated excellent relevance and content coverage, yielding S‐CVI/Ave values of 0.97 for knowledge and 0.99 for practice, significantly exceeding the 0.90 threshold for clinical tool validation.

While formal construct validity via factor analysis was not conducted due to the study’s primary focus on intervention efficacy, the instrument’s validity was ensured through rigorous alignment with the CDC and WHO evidence‐based guidelines for infection control in hemodialysis. This ensured that the items accurately represented the essential clinical domains required for specialized nursing practice. Additionally, face validity was confirmed during the pilot phase, where 90% of participants reported that the items were clear and relevant to their daily clinical duties.

The reliability of the instruments was measured using internal consistency following the pilot phase, demonstrating Cronbach’s alpha values of 0.86 for the knowledge test and 0.82 for the practice scale. These values indicated good to excellent reliability for the instruments in this clinical context.

### 2.10. A Pilot Study

A pilot test was also conducted on 10 hemodialysis registered nurses to assess the clarity, feasibility, and reliability of the study tools as well as the time required for data collection. The participants in the pilot study were excluded from the main study sample. It was notable that a very high proportion of participants (90%) mentioned that the questionnaire items were clear and easy to understand. Ten percent of them proposed constructive comments regarding the questionnaire’s wording. The researchers used 20 min of data collection time for each participant, which lasted between 18 and 25 min because they found this period suitable for medical environments. Most of them reported that the chatbot intervention was a feasible tool that all nurses could use with ease, accepting the ease of the program. They liked being able to access the program conveniently. Some participants noted that further modifications should be made to the online chatbot in order to make it more interactive and improve retention of information. According to their feedback, the questionnaire was revised by changing some wording, and more interactive activities were added.

### 2.11. Study Procedure

The study protocol received formal approval from the IRB at Jordan University of Science and Technology (ID: 10/166/2024). Administrative approvals were obtained from the relevant hospital administrations and the head nurses of all participating clinical units.

The study used a cluster‐randomized design to meet the logistical needs of the clinical environment. First, the cluster randomization was conducted using the combination of hospital site and work shift (Shift A vs. Shift B) as the unit of randomization. Essentially, five of the participating hospitals produced ten distinct clusters. The process of allocation was carried out by a researcher who was not involved in data collection and/or intervention delivery. This researcher randomly distributed (equally) the ten clusters between the experimental and control groups using a computer‐generated random‐number sequence. Five clusters were allocated to each group, revealing 25 participants allocated per group. All cluster assignments were made and secured in advance before the start of the recruitment and baseline data collection.

Blinded recruitment was then conducted in the ten clusters employing a convenience sampling technique. The enrolment of five registered nurses per cluster was targeted using a fixed sampling frame, which was designed by the principal investigator in collaboration with the administrations of the participating hospitals. An independent researcher, who was kept strictly blinded to the randomization sequence, approached all eligible staff during shift changes to conduct enrollment. This independent researcher individually approached nurses in their respective clinical settings, explained the objectives of the study, and informed them of the eligibility criteria. The nurses who met the inclusion criteria were invited to give written informed consent to participate. The recruitment proceeded until the chosen total sample size of fifty participants (*n* = 25 in each group) was reached. This independent researcher was kept strictly blinded to the cluster assignments (experimental and control) during the entire enrolment and consent process. Statistical analyses verified that using these randomization and recruitment approaches led to the creation of two homogeneous groups, which did not differ significantly in terms of demographic or professional variables (*p* 0.05).

The research team employed various protocols in order to reduce contamination in the study. First, the cluster‐level randomization was used, i.e., all nurses working at the same location and shift were given the same allocation. Second, the participants of the experimental group were clearly asked not to disclose any data about chatbots. Third, members of the experimental group received unique access to the intervention through personal WhatsApp accounts.

Baseline measurements were carried out right after enrolment, but strictly preceding any intervention given by a blinded research assistant who was not aware of the cluster allocations. More importantly, at this point, all participants did not know their particular group assignment, thereby reducing expectation bias. The same blinded assistant implemented the posttest 28 days (4 weeks) after the 5‐day intervention period. This timeframe ensures that the study results reflect sustained learning and behavioral modification—demonstrating the “Transfer of Learning”—rather than mere immediate posttraining recall. This longitudinal approach to assessment aligns with previous research and established methodologies in healthcare education research [36, 38].

### 2.12. Ethical Considerations

The study received ethical approval from the IRB of Jordan University of Science and Technology (IRB approval number: 10/166/2024). Participants provided written informed consent after receiving a general explanation of the study objectives and instructions on using the chatbot via the WhatsApp application interface. Participants were informed that they could decline participation or discontinue involvement in the study at any point without any negative consequences and that their data would be kept confidential and anonymized. To maintain confidentiality, the study data were stored in a password‐protected computer with numbers replacing the participants’ names.

### 2.13. Data Analysis

The study data were analyzed using the Statistical Package for Social Sciences (SPSS Version 25.0). The assumptions of the statistical tests were checked to ensure that the analyses were valid. Normality was checked by histogram graphs, Shapiro–Wilk test, and values of skewness and kurtosis. The homogeneity of variance was tested using Levene’s test. The equivalence of the two groups was assessed with respect to demographic characteristics and baseline values of the dependent variables, using independent *t*‐tests and chi‐square tests according to the measurement level of each variable. For the “Level of Education” variable, where more than 20% of the cells had an expected frequency of less than five, Fisher’s exact test was employed to provide a more robust and accurate *p* value.

The main analysis was conducted using 2 × 2 mixed‐model RM ANOVA tests to detect any statistically significant interaction effect between the two study groups across the two measurement times. To determine the statistical significance of the results, *p* values of 0.05 or less were utilized for all statistical tests conducted in the study.

## 3. Result

### 3.1. Demographic Characteristics and Baseline Differences in Demographic

Table 1 shows the demographic characteristics of the study sample. Based on the chi‐square test, the results revealed that the two groups were not significantly different in terms of sex (*x* ^2^ (1) = 0.56, *p* = 0.451), education training (*x* ^2^ (1) = 0.00, *p* = 1.00), and level of education (Fisher’s exact test, *p* = 0.667). The *t*‐test showed that the two groups were also not significantly different in terms of age (*t* (48) = −1.53, *p* = 0.13), clinical experience in hemodialysis unit (*t* (48) = −0.38, *p* = 0.705), baseline infection control knowledge (*t* (48) = 0.07, *p* = 0.942), and baseline infection control practice (*t* (48) = 0.29, *p* = 0.772) (see Table 1).

**TABLE 1:** Baseline demographic and outcome characteristics of study groups (*N* = 50).

Characteristic	Experimental group Frequency (%)/mean (SD)	Control group Frequency (%)/mean (SD)	Test statistic *x* ^2^/t‐value (*p* value)
*Sex*			
Male	12 (48)	9 (36%)	0.56 (0.451)
Female	13 (52)	16 (64%)	

*Level of education in nursing*			
Diploma	1 (4)	0 (0%)	
Baccalaureate	22 (88)	21 (84%)	^a^(0.667)
Master	2 (8)	4 (16%)	

*Education training*			
Yes	16 (64)	16 (64%)	0.00 (1.000)
No	9 (36)	9 (36%)	
Age (years)	36.92 (7.15)	36.08 (5.85)	−1.53 (0.130)
Hemodialysis experience (years)	12.08 (6.51)	11.42 (5.70)	−0.38 (0.705)
Baseline knowledge (score range 0–33)	18.64 (5.39)	18.76 (6.09)	0.07 (0.942)
Baseline practice (score range: 48–240)	204.00 (20.93)	205.00 (23.61)	0.29 (0.772)

*Note:* *x* ^2^ *= *chi‐square test.

Abbreviation: SD = standard deviation.

^a^ *p* value calculated using Fisher’s exact test for the level of education variable due to expected cell frequencies < 5.

### 3.2. Descriptive Statistics for Infection Control Knowledge and Practice

Descriptive statistics for knowledge scores are presented in Table 2. The experimental group demonstrated an increase in the infection control and practice knowledge mean scores, while the control group demonstrated a decrease in the infection control and practice knowledge mean scores from pretest to posttest.

**TABLE 2:** Descriptive statistics for knowledge and practice scores by group and time.

Group	Variable	Time	Minimum	Maximum	Mean (SD)
Control (*n* = 25)	Knowledge (score range: 0–33)	Pretest	6.00	28.00	18.76 (6.09
		Posttest	10.00	27.00	16.92 (4.51)
	Practice (score range: 48–240)	Pretest	156.00	240.00	205.84 (23.61)
		Posttest	176.00	232.00	200.84 (16.46)
Experimental (*n* = 25)	Knowledge (score range: 0–33)	Pretest	6.00	26.00	18.64 (5.39)
		Posttest	16.00	32.00	24.96 (4.01)
	Practice (score range: 48–240)	Pretest	160.00	233.00	204.00 (20.93)
		Posttest	198.00	240.00	221.60 (11.88)

Abbreviation: SD = standard deviation.

### 3.3. Effect of Training on Infection Control Knowledge and Practice

As presented in Table 3, a 2 × 2 mixed‐model RM ANOVA was conducted to assess the impact of chatbot‐based training, compared to the control condition, on infection control knowledge and practice among hemodialysis nurses.

**TABLE 3:** Analysis of variance (ANOVA) on knowledge and practice scores across two time points by group.

Variable	Time effect *F* (*p* value)	Group effect *F* (*p* value)	Interaction effect *F* (*p* value)
Knowledge	5.83 (*p* = 0.020)	13.11 (*p* = 0.001)	19.34 (*p* < 0.001)
Practice	4.51 (*p* = 0.039)	4.61 (*p* = 0.037)	14.52 (*p* < 0.001)

### 3.4. Infection Control Knowledge

First, the assumption of homogeneity of variance was assessed using Levene’s test. The results were nonsignificant for the infection control knowledge at both the pretest (*p* = 0.563) and posttest (*p* = 0.722), confirming that the assumption of homogeneity of variance was met. The 2 × 2 mixed‐model RM ANOVA results showed a significant main effect of time (*F* (1, 48) = 5.832, *p* = 0.02, partial *η* ^2^ = 0.108), indicating an overall increase in knowledge scores from pretest to posttest. Also, there was a significant main effect of group (*F* (1, 48) = 13.11, *p* = 0.001, partial *η* ^2^ = 0.21), suggesting that the experimental group had higher overall knowledge scores than the control group. Notably, a significant interaction effect was observed between group and time (*F* (1, 48) = 19.34, *p* < 0.0001), with a large effect size (partial *η* ^2^=0.28), demonstrating that the increase in infection control knowledge scores from pretest to posttest was significantly greater in the experimental group compared to the control group.

Simple effects analysis revealed that the experimental group demonstrated a significant increase in infection control knowledge from the pretest (*M* = 18.64, SD = 5.39) to the posttest (*M* = 24.96, SD = 4.01), *p* < 0.0001. In contrast, the control group did not significantly change in infection control knowledge scores from the pretest (*M* = 18.76, SD = 6.09) to the posttest (*M* = 16.92, SD = 4.51), *p* = 0.21.

### 3.5. Infection Control Practice

First, Levene’s equality test was nonsignificant for the infection control practice at the pretest (*p* = 0.323) and at the posttest (*p* = 0.09), indicating that the assumption of homogeneity of variance was met. The overall practice score ranges from 48 to 240, representing the 48‐item scale evaluated with a 5‐point Likert system. Such a large range was purposefully used in the attempt to give the granularity needed to capture slight behavioral changes across the complex clinical areas of infection control.

For infection control practice, the mixed‐model RM ANOVA results showed that there was a significant main effect of time (*F* (1, 48) = 4.513, *p* = 0.039, partial *η* ^2^ = 0.086), indicating an overall increase in infection control practice scores from pretest to posttest. Also, there was a significant main effect of group (*F* (1, 48) = 4.61, *p* = .037, partial *η* ^2^ = 0.08), suggesting that the experimental group had higher overall infection control practice scores than the control group. Most importantly, there was a significant interaction effect between group and time (*F* (1, 48) = 14.52, *p* < 0.0001), with a large effect size (partial *η* ^2^ = 0.23), indicating that the increase in infection control practice scores from pretest to posttest was significantly greater in the experimental group compared to the control group.

Simple effects analysis revealed that the experimental group demonstrated a significant increase in infection control practice from the pretest (*M* = 204.00, SD = 20.93) to the posttest (*M* = 221.60, SD = 11.88), *p* < 0.0001. In contrast, the control group showed a nonsignificant decrease in ICP practice scores from the pretest (*M* = 205.84, SD = 23.61) to the posttest (*M* = 200.84, SD = 16.46), *p* = 0.227.

## 4. Discussion

The principal finding of this cluster‐randomized controlled trial was the significant positive effect of the WhatsApp chatbot‐based training program on both infection control knowledge and practices among Jordanian hemodialysis nurses, evidenced by strong Group × Time interaction effects. The intervention produced large effect sizes, which reached 0.28 for knowledge and 0.23 for practice, thus demonstrating its significant clinical value. However, these practice improvements should be interpreted with caution. Since practice was assessed via self‐report, it may have been affected by social desirability bias, in which participants report greater adherence to sterile practices to meet the perceived research expectations rather than reflecting actual bedside practice. Consequently, although the chatbot helped reinforce nurses’ understanding of the theoretical steps of infection control, future research should also include direct observational audits. This would help determine whether the improvements reported by participants are truly reflected in their actual clinical practice, such as actual hand‐hygienic practice.

The control group showed a nonsignificant reduction in infection control knowledge (18.76–16.92) and practice scores (205.84–200.84) between baseline and posttest results. These findings revealed that the control condition was not sufficient to trigger a significant change, highlighting the experimental intervention’s efficacy. The slight downward trend in the control group may be attributed to the natural decay of knowledge in the absence of reinforcement. Crucially, because the control group did not receive any standard educational materials, structured training, or placebo interventions during the 28‐day period, this decline suggests that routine clinical practice alone—without active pedagogical reinforcement—is insufficient to maintain or improve complex infection control competencies. In addition, this small decrease may possibly be explained by other external conditions, including variable clinical workloads, environmental stress, or inadequate cognitive priming. Ultimately, the significant Group × Time interaction underscores the success of the intervention. While the control group remained largely stagnant, the experimental group demonstrated substantial and statistically significant improvements.

The effectiveness of the study intervention is potentially attributed to its adherence to the principles of learning theories [39]. The WhatsApp chatbot training model implemented constructivist concepts, thus transforming nurses into active, autonomous learners [39]. The main mechanism was the use of a scenario‐based case discussion with combined and immediate knowledge testing, a design that forced participants to use their knowledge and build understanding in a situation resembling real‐world clinical decision‐making. Furthermore, the feedback loop was vital because of its immediacy and personalization. In case nurses made a mistake, the model provided a brief explanatory fact and referred them to the relevant material. This instantaneous dynamic effectively seals the knowledge gap, helps in dispelling misconceptions, and revises the procedural memory—a recognized trigger of skill acquisition and behavioral modification, which the traditional, noninteractive form of training cannot emulate [23, 24].

A critical distinction of our model lies in the choice of delivery platform and instructional architecture, which helps explain how our findings extend those reported in prior studies. Previous literature evaluating standalone chatbot applications has demonstrated improvements in knowledge and practice across clinical domains; however, these interventions often report challenges related to engagement and retention, largely due to the need to navigate unfamiliar digital environments [19, 20, 22–25, 39, 40]. In contrast, by integrating the intervention within WhatsApp, our study likely reduced these technological and cognitive barriers, which may explain the higher adherence and stronger learning effects observed. Similarly, while earlier studies using WhatsApp have typically relied on unstructured communication approaches, limiting standardization and instructional consistency [17, 18], the present study employed a deterministic, rule‐based chatbot that ensured uniform delivery of evidence‐based procedural content. This distinction is particularly important when interpreting our findings, as standardized, stepwise instruction is essential for developing complex infection control competencies. Furthermore, although prior research has established the feasibility of chatbot‐mediated learning, most studies have been conducted in general or academic settings and have not examined performance within high‐acuity clinical environments [19, 20, 22]. In contrast, the current study was conducted in hemodialysis units, where the alignment between scenario‐based training and real clinical demands may have enhanced situated learning and facilitated the translation of knowledge into practice. Taken together, these differences suggest that the stronger effects observed in this study are not only consistent with previous findings but are also likely amplified by the combined influence of platform familiarity, deterministic instructional design, and the high‐acuity clinical context.

Building on these findings, the practical implications of this approach are particularly noteworthy. Consequently, this study addresses key limitations of traditional educational delivery, particularly high costs and scheduling constraints [40]. The intervention’s simple, 5‐day asynchronous structure enabled implementation independent of resource‐intensive, place‐based training models. This flexibility allowed routine clinical workflows to remain undisturbed [23, 24]. The high compliance and improved clinical outcomes observed in this study highlight the feasibility and effectiveness of low‐friction, AI‐supported e‐learning models for nurses in high‐risk clinical settings [23–25, 41].

### 4.1. Implications for Nursing Management

The empirical results of the present research suggest that the intervention contributed to improvement in both theoretical and practical skills associated with infection control in hemodialysis settings. The results support the assumption that training conducted through a chatbot based on WhatsApp is an evidence‐based pedagogy modality that can be potentially used in ongoing professional development. The findings also provide preliminary evidence that asynchronous digital education may act as an effective alternative to face‐to‐face education. The methodological framework ascribes to the institutions a feasible plan of taking advantage of combined data recording to attain real‐time supervision of compliance, coupled with prudent allocation of resources. Furthermore, this model may facilitate the achievement of targeted clinical outcomes through a cost‐effective framework, offering a potential template that could be adapted to support improved safety standards in various resource‐limited healthcare settings. While these findings are promising, further research across diverse clinical environments is necessary to confirm the long‐term scalability and impact of such digital interventions.

### 4.2. Limitations

Limitations of this study may include the following: First, generalizability was potentially compromised by the fact that the findings referred specifically to nurses in hemodialysis units. Therefore, the results could not be generalized to other healthcare settings or professionals. Second, although the randomization of clusters in the study sites enhances internal validity, the selection of the participating hospitals and participants through convenience sampling is also a weakness. Nonrandom selection of such facilities combined with possible selection bias among research participants can limit the generalizability (external validity) of the results to the overall population of hemodialysis units in Jordan or other regions. Third, since the measure of practice was conducted through self‐report, it might be subject to social desirability bias, where respondents indicate a higher level of adherence than would be demonstrated during a clinical performance. Although there were a number of strategies implemented that minimized these adverse effects (including objective chatbot logging to capture real‐time attendance, full participant anonymity, and a standardized 28‐day follow‐up protocol), future research ought to incorporate direct observational audits to prove these perceived competencies. Fourth, despite using strategies to avoid contamination, such as cluster randomization, explicit confidentiality instructions, and restricting access via unique, individualized login credentials, the possibility of this threat remains a key limitation inherent in RCT designs, as nurses from both groups worked in the same hospitals. Finally, the relatively short follow‐up period of 4 weeks limits the ability to draw conclusions regarding the long‐term retention of infection control knowledge and the sustainability of behavioral changes over time.

### 4.3. Recommendations for Future Research

To expand the perspectives of future investigations, chatbots can be implemented to conduct research involving more diverse and bigger cohorts to be able to generalize the findings to a range of situations and populations. Longitudinal studies are necessary to determine the lasting impact of e‐learning‐based education interventions on infection control, which will provide a useful insight into the progressive change in the knowledge and practices of nurses during a prolonged temporal context. With the ongoing development of artificial intelligence, introducing intelligent systems in the form of chatbots as dynamic coaching agents in research projects will also contribute to real‐time learning and support, which introduces a new direction in lifelong learning and further promotes the development of healthcare education. The use of objective measures instead of self‐report items in the evaluation of infection control practices is expected to produce more reliable and valid results, as response biases such as recall errors and social desirability effects will skew the results.

## 5. Conclusion

The research aimed to examine how WhatsApp chatbot‐based educational training affects Jordanian nurses’ infection knowledge and practice in Jordanian hemodialysis units. The experimental group showed better knowledge and application of infection control practices than the control group after finishing the educational training program, according to ANOVA results, which demonstrated substantial interaction effects. This pedagogical model supports sustainable professional development and reinforces continuous learning by providing a flexible, asynchronous platform for ongoing competency maintenance. Ultimately, this study validates a theory‐grounded digital framework that effectively bridges the gap between theoretical guidelines and clinical practice, offering a scalable solution for nursing education in high‐acuity, resource‐limited settings.

## Funding

This study was funded by the Deanship of Research at Jordan University of Science and Technology (Research Grant No. 20240058). The funding does not cover the article processing charge (APC).

## Conflicts of Interest

The authors declare no conflicts of interest.

## Data Availability

The data that support the findings of this study are available on request from the corresponding author. The data are not publicly available due to privacy or ethical restrictions.
